# Is graphite lithiophobic or lithiophilic?

**DOI:** 10.1093/nsr/nwz222

**Published:** 2020-01-03

**Authors:** Jian Duan, Yuheng Zheng, Wei Luo, Wangyan Wu, Tengrui Wang, Yong Xie, Sa Li, Ju Li, Yunhui Huang

**Affiliations:** Institute of New Energy for Vehicles, School of Materials Science and Engineering, Tongji University, Shanghai 201804, China; Institute of New Energy for Vehicles, School of Materials Science and Engineering, Tongji University, Shanghai 201804, China; Institute of New Energy for Vehicles, School of Materials Science and Engineering, Tongji University, Shanghai 201804, China; Institute of New Energy for Vehicles, School of Materials Science and Engineering, Tongji University, Shanghai 201804, China; Institute of New Energy for Vehicles, School of Materials Science and Engineering, Tongji University, Shanghai 201804, China; Institute of New Energy for Vehicles, School of Materials Science and Engineering, Tongji University, Shanghai 201804, China; Institute of New Energy for Vehicles, School of Materials Science and Engineering, Tongji University, Shanghai 201804, China; Department of Nuclear Science and Engineering and Department of Materials Science and Engineering, Massachusetts Institute of Technology, Cambridge, MA 02139, USA; Institute of New Energy for Vehicles, School of Materials Science and Engineering, Tongji University, Shanghai 201804, China

**Keywords:** wetting, contact-line hysteresis, surface-pinning defects, electrochemical-stability windows, Li–graphite composite

## Abstract

Graphite and lithium metal are two classic anode materials and their composite has shown promising performance for rechargeable batteries. However, it is generally accepted that Li metal wets graphite poorly, causing its spreading and infiltration difficult. Here we show that graphite can either appear superlithiophilic or lithiophobic, depending on the local redox potential. By comparing the wetting performance of highly ordered pyrolytic graphite, porous carbon paper (PCP), lithiated PCP and graphite powder, we demonstrate that the surface contaminants that pin the contact-line motion and cause contact-angle hysteresis have their own electrochemical-stability windows. The surface contaminants can be either removed or reinforced in a time-dependent manner, depending on whether the reducing agents (C_6_→LiC_6_) or the oxidizing agents (air, moisture) dominate in the ambient environment, leading to bifurcating dynamics of either superfast or superslow wetting. Our findings enable new fabrication technology for Li–graphite composite with a controllable Li-metal/graphite ratio and present great promise for the mass production of Li-based anodes for use in high-energy-density batteries.

## Teaser

Contact-line hysteresis is observed in liquid lithium wetting of graphite, depending on surface pinning defects and oxidizing agents (air, moisture) in the ambient environment, with implications for the compositing of these two classic anode materials

## INTRODUCTION

Lithium metal is the ‘holy grail anode’ for rechargeable batteries due to its low potential (−3.04 V vs. standard hydrogen electrode) and high theoretical specific capacity (3861 mAh/g) [1–5]. However, challenges due to unstable solid electrolyte interphase, non-uniform deposition, dendritic penetration and volume change have hindered its application [6–9]. Much effort has been dedicated to making improvements, such as electrolyte optimization [10–12] and interfacial engineering [13]. Another attractive route for addressing these problems is to manufacture a Li-metal–matrix composite anode by incorporating body-centered cubic Li metal (Li_BCC_) into a conductive matrix, which can maintain a stable volume and shape upon repeated electrochemical cycling [14–17].

Various carbonaceous hosts including reduced graphene oxide [18], porous graphene networks [19] and porous carbon film [20] have been investigated as the matrices to host Li_BCC_. To fabricate the composite, the wettability between molten Li (Li_liq_) and graphite matrix is crucial. Unfortunately, it was generally accepted that graphite is lithiophobic and Li_liq_ shows poor spreading on a graphite surface [21]. Recent studies have suggested that coating a layer of Li-reactive material such as Si and the interfacial reaction between Li and the coating material drive the lithiophobic-to-lithiophilic transition [22,23]. However, such a change in liquid-spreading behavior is due to the replacement of the graphite by the reactive coating. Consequently, it might be asked whether graphite is intrinsically lithiophobic or lithiophilic.

We note three conceptual subtleties with the posing of this question. First, textbook Young's equation is defined for immiscible or *non-reactive* bulk phases α (solid substrate), β (Li_liq_) and δ (vapor):

(1) \begin{equation*}{{\rm{\gamma\! }}_{{\rm{\alpha \beta }}}} + {{\rm{\gamma }}_{{\rm{\beta \delta }}}}\cos \,{{\rm{\theta }}_{{\rm{\alpha \beta }}}} = {{\rm{\gamma }}_{{\rm{\alpha \delta }}}},\end{equation*}

when no bulk phase change is happening and the only thing that could change is the physical interfacial contact line controlled by the interfacial tension forces γ_αβ_, γ_βδ_ and γ_αδ_ [24]. In a so-called ‘reactive-wetting’ scenario, however, the bulk phases themselves (e.g. the substrate α) could be undergoing bulk reactions. One can still measure an apparent contact angle (ACA) θ_αβ_ at a certain time *t* at a certain observation-length scale, but if α is changing with time, so can θ_αβ_(*t*). Since bare graphite (C_6_) reacts with Li_liq_: C_6_ + Li_liq_ = LiC_6_ as a bulk phase change, with a *bulk* Gibbs-free energy difference, reactive wetting is also an issue here, just as with a Si coating. Second, we note that, generally speaking, as lithiation proceeds, the absolute electrochemical potential of the substrate *U*^α^ changes and certain surface chemical groups that were thermodynamically stable at previous *U*^α^ may cease to be; for example, O^*^ (where ^*^ stands for surface site on α) may turn into (Li*_x_*O)_n_^*^ clusters. This means that, even if there was *no* bulk phase transition, the equilibrium θ_αβ_^eq^_  _should still be a function of *U*^α^. Thus, instead of asking ‘what is the wetting angle of β on α?’, the more appropriate question should be ‘what is the wetting angle of β on α at *that* potential?’. Third, there are issues of kinetics (not just thermodynamics), fluid dynamics and geometry, when talking about the phenomenology of the β phase physically spreading on or infiltrating into α. Because the interfacial tensions of Eq. (1) are physically driving the convections [25] and because the β-phase-geometry changes also affect the mass transport and later bulk phase change as well, this causality chain affects how the β phase moves through the potentially intricate geometries of α. If there are certain slow steps in the causality chain (e.g. bulk diffusion or electron-tunneling induced surface chemical group decomposition [26]), then it could take significantly longer to cover so much area of a porous substrate than a fully dense, non-porous substrate.

**Figure 1.**
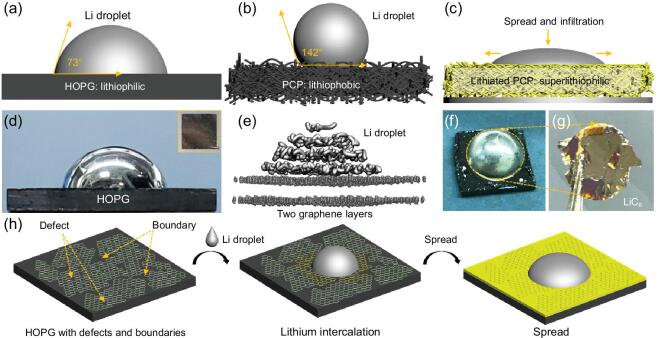
Li wettability with graphitic carbon substrates. Schematic of ACA measurement of liquid Li droplet on (a) HOPG and (b) PCP. (c) A wettability transition from lithiophobic to superlithiophilic of PCP occurs due to the Li intercalation into the PCP and phase change from C_6_ to LiC_6_. Photos of liquid-Li droplet on (d) HOPG and inset is top view of HOPG. (e) *Ab initio* MD calculation of a Li droplet/HOPG system at 500 K. (f, g) Top view of the liquid-Li droplet on the HOPG after cooling down to room temperature and a few lithiated graphite layers from HOPG still adhered to Li after peeling off the Li droplet from the HOPG. (h) Schematic of the mechanism for spreading the Li droplet on the HOPG.

To address these questions, we conducted ACA measurements with a molten Li_liq_ drop on highly ordered pyrolytic graphite (HOPG), which is fully dense, and also on porous carbon paper (PCP), which is porous, inside a glovebox. We observed that the HOPG substrate immediately allows an ACA of 73° with Li_liq_ (Fig. 1a). This is the first demonstration, to our knowledge, that graphite can be considered ‘lithiophilic’, which is encouraging from the view of fabricating Li-metal–matrix composite by Li_liq_ infiltration. However, when placing a molten Li_liq_ drop on PCP in the glovebox, the ACA is as large as 142° (Fig. 1b) and Li_liq_ infiltration does not appear to happen at all on the timescale of 10^3^ seconds. There may be two possible explanations for this: (i) although the PCP fibers consist of largely graphitic carbon, the composition and thermodynamics could be somewhat different from those of HOPG; (ii) kinetics could be in play here: there could be certain impediments (surface contaminants) to the Li_liq_ phase infiltrating the pores, pinning the contact lines, and, if it takes too long to infiltrate, the trace O_2_, CO_2_, etc. in the glovebox could become new impediments to form a solid oxidation layer on the Li_liq_ surface that further slows down the infiltration. After careful analysis and modeling, we have determined that (ii) is the likely cause.

To show this, we first pre-lithiate PCP to PCP_lithiated_ with almost equal porosity. This makes the substrate much more reducing electrochemically. We find that, while PCP exhibits apparent lithiophobicity, the same porous paper structure changes to superlithiophilic upon the phase change from C_6_ to LiC_6_ (Fig. 1c). This means the viscosity of Li_liq_ and the pore diameter of PCP cannot be the limiting factors to infiltration; instead, the C_6_ → LiC_6_ phase change and the *U*^α^-dependent θ_αβ_^eq^ could be the cause here due to the fact that some surface chemical groups (surface contaminants) pinning the contact line could be removed at *this* potential. Based on our findings, a new liquid-metal-infiltration fabrication process for Li–PCP composite has been developed with a controllable Li/PCP mass ratio. Encouragingly, a large piece of Li–PCP composite anode was successfully prepared and showed excellent performance in pouch-type Li–S cells.

## RESULTS AND DISCUSSION

### ACA observations on HOPG and PCP

Two types of carbonaceous substrates were adopted to conduct the ACA measurements. The first one is HOPG due to its high chemical purity, high density, ultra-flat surface and high degree of graphitization (Supplementary Fig. 1). The as-received HOPG was first cleaned by the well-known ‘Scotch Tape’ method, which has been widely employed to prepare single- or few-layer graphene [27]. HOPG exhibits a flat and shiny surface after the ‘Scotch Tape’ cleaning (inset of Fig. 1d) and XPS results further reveal a clean surface without functional organic groups, such as –OH or C = O (Supplementary Fig. 2). A Li_liq_ droplet was then deposited on the HOPG surface, while the HOPG was placed on a hot plate with a temperature of ∼225°C. The whole process can be seen in Supplementary Movie 1. Surprisingly, the ACA is as small as 73° (Fig. 1d).

To check this experiment against theory, an *ab initio* molecular dynamics simulation was performed with a molten Li droplet (54 Li atoms)/graphite (432 C atoms, two-layered graphene) setup to prove that a clean (002) surface of graphite is intrinsically lithiophilic at 500 K. Figure 1e presents the trajectories of the Li and carbon atoms of the last 1000 steps of the simulation. As previously discussed, this system consists of the α (graphite), β (Li_liq_) and δ (lithium vapor phase) phases, although the equilibrium vapor pressure of Li is extremely low at this temperature. The C atoms of graphite are allowed to vibrate, which presents a *c*-direction preference, but, interestingly, the Li_liq_ exhibits a layered-like atomic structure near the contact interface even though in a liquid state. The highly delocalized ${\rm{\pi }}$-bonds of graphene enable Li atoms to wander around, but they still bind together due to the strong Li–Li affinity, which implies a more ‘physical’ character than chemical bonding for the Li atoms in contact with the graphene sheet. The ACA is observed to be ∼62°, which is similar to what we observed in the HOPG experiment above. The Li/lithiated graphite configuration was also investigated in the same manner with *ab initio* molecular dynamics (MD), which possess almost the same ACA, as shown in Supplementary Fig. 3. This confirms that both C_6_ and LiC_6_ are intrinsically (thermodynamically) lithiophilic and prefer to be enveloped by Li_liq_ rather than the vapor phase, when having a clean surface.

Experimentally, upon removing the Li droplet@HOPG from the hot plate, the Li droplet solidified quickly. From the top view, it is clear that Li has spread on the HOPG surface, as outlined by the dotted line in Fig. 1f. Interestingly, Li_BCC_ glued so firmly onto the HOPG that it was difficult to separate the Li_BCC_ from the HOPG. We had to forcefully peel off the Li_BCC_ from the HOPG using tweezers while the top layers of the HOPG were still adhered to the Li_BCC_ (Supplementary Movie 2). The adhered layers showed a brilliant yellow color (Fig. 1g), suggesting the formation of LiC_6_: the stage-1 Li–graphite intercalation compounds (Li–GICs). This phenomenon corresponds to the intercalation of Li into graphite layers in the interior of the HOPG. We propose that HOPG is intrinsically lithiophilic and Li can penetrate the graphite layers underneath the top surface of HOPG through the grain boundaries and/or in-plane defects of the graphite layers and lead to a spontaneous Li intercalation process, which further promotes the spreading of Li_liq_ on HOPG, as schematically described in Fig. 1h.

### Transition from lithiophobicity to superlithiophilicity of PCP and graphite powder

**Figure 2.**
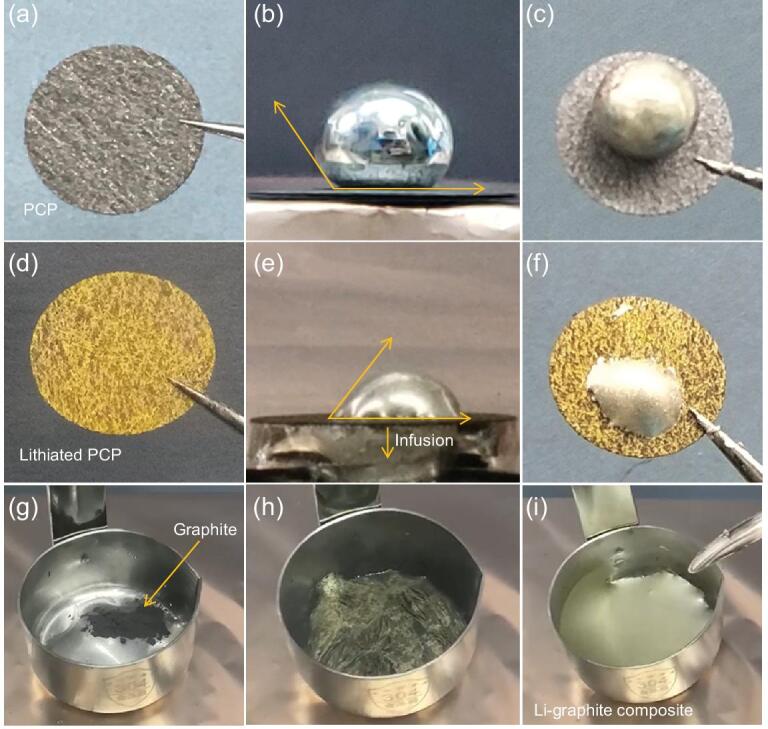
Li wettability of PCP, lithiated PCP and graphite powder. ACA measurements of (a, b) PCP and (d, e) lithiated PCP. Digital photos of a Li droplet on (c) PCP and (f) lithiated PCP after cooling down to room temperature. The infiltration and penetration of the Li droplet into the lithiated PCP framework occur in 2 seconds. (g–i) The process of mixing Li and graphite powder under mechanical stirring in which the stirring plays a critical role in promoting the lithiation and uniform distribution of lithiated graphite in Li.

Our conclusion that graphite is intrinsically lithiophilic seems to contradict with previous experiments that found Li_liq_ cannot wet PCP [22]. This prompted us to gain further understanding of the effect of surface chemistry, substrate topographic features and competing reactive dynamics (C_6_ → LiC_6_ together with Li_liq_ → Li_2_O/Li_2_CO_3_). As shown in Fig. 2a and b, the ACA with PCP is as large as 142° (measurement process shown in Supplementary Fig. 4 and Supplementary Movie 3), even though PCP consists of mainly graphitic carbon fibers (XRD patterns in Supplementary Fig. 5). We then moved Li droplet@PCP from the hot plate and observed that the solidified Li on the PCP is not as ‘shiny’ as the starting droplet (Fig. 2c). We then ion-milled the surface of the lithium droplet to measure the depth of the impurities. It turned out that the impurity of Li_2_O can still be detected even after ion-milling 200 nm away, indicating a strong reaction of Li_liq_ with trace impurities in the glovebox (Supplementary Fig. 6). A trace amount of air in the glovebox and functional groups on the PCP would react with Li_liq_ and form Li oxides, carbonates or nitrides. These products can slow down the Li intercalation into PCP by the blockage of Li^+^ or electron transfer, which act as wetting barriers on the PCP surface and enhance the lithiophobic behavior.

We then studied the spreading behavior of the Li droplet on the pre-lithiated PCP. The pre-lithiation was conducted by floating the PCP on top of molten Li_liq_ to increase the contact area. The gravity and inertia of a small piece of PCP floating on top of a large liquid-metal pool were sufficient to break the Li_2_O/Li_2_CO_3_ scale. As expected, PCP gradually became yellow and a piece of lithiated but still porous PCP_lithiated_ was obtained (Supplementary Movie 4 and Fig. 2d). XRD measurement confirmed that LiC_6_ was the main phase in PCP_lithiated_ (Supplementary Fig. 7). ACA measurement was then carried out by depositing another Li_liq_ drop onto the new surface of PCP_lithiated_. Surprisingly, a much smaller ACA was found compared to pristine PCP. Moreover, Li infused into the pores and even penetrated through the whole porous substrate extremely rapidly, in 2 seconds (Fig. 2e and f, and Supplementary Movie 5). All the observations thus supported the conclusion that PCP changed from lithiophobic to superlithiophilic due to the phase change (C_6_→LiC_6_) and prevalence of the chemically reducing party, even though the bulk surface morphology of LiC_6_ is still rougher than HOPG. As evidenced by the XPS result of PCP (Supplementary Fig. 8), 10% of the surface was covered by O-containing groups. Some surface defect groups would surely become unstable as *U* → 0 versus Li^+^/Li, and can be transformed in a time-dependent manner. The whole transition process and infiltration of Li_liq_ into PCP_lithiated_ are schematically illustrated in Supplementary Fig. 9.

Graphite powders were further adopted to investigate the Li–graphite wettability (Supplementary Fig. 10). When initially sprinkled onto the Li_liq_ surface, just like the PCP, the graphite powders floated (Fig. 2g), showing apparent lithiophobicity. But, under mechanical stirring, the Li_2_O/Li_2_CO_3_ scales broke and the graphite powder gradually turned bright yellow (Fig. 2h), and then the lithiated graphite powders were sucked into the Li_liq_ bulk. The surface color thus changed sequentially from black (sprinkled C_6_) → bright yellow (sprinkled LiC_6_) → silver (LiC_6_ pulled inside, with Li_liq_ back on top, Supplementary Fig. 11), and the lithiated graphite powders could be uniformly dispersed into the liquid Li (Fig. 2i and Supplementary Movie 6), which is further revealed by the scanning electron microscopy (SEM) images and its corresponding EDX (Supplementary Fig. 12), indicative of a lithiophobicity–superlithiophilicity transition by Li intercalation. In contrast, without stirring, the lithiation and dispersion of the graphite powder were much slower.

The keys to the seemingly paradoxical reactive-wetting behavior are thus the competition between *two* reactions: α-side reducing C_6_ → LiC_6_ and associated surface-chemistry change versus β-side oxidative Li_liq_ → Li_2_O/Li_2_CO_3_, in a non-ideal environment with oxidative gaseous species. Starting with the initial PCP, certain α-side surface impurities or chemical groups can pin the contact line, causing contact-line hysteresis. (But these surface chemical groups are not immortal and must have their own electrochemical-stability windows, like any electrolyte molecules or salt anions [26].) A slower Li_liq_ spreading due to the ‘dirtier’ surfaces of PCP compared to HOPG with more pinning sites [28], plus the longer distance to cover due to the porous geometry of PCP, cause the competing reaction Li_liq_ → Li_2_O/Li_2_CO_3_ to catch up, forming nonconductive solid oxide scales covering Li_liq_ that impede further spreading and intercalation. We note that, at ∼225°C and *U*∼0 V, Li_liq_ and even lithiated PCP are very reducing and super-attractive to mobile O_2_, CO_2_ gases. Given enough time, in practical experiments, the formation of oxide/carbonate scales on Li_liq_ and even the lithiated PCP surface is *inevitable*. The only way to overcome such a defense is for Li_liq_ to rush in quickly enough and expel the O_2_/CO_2_-containing δ (vapor) phase completely (defined as ‘*Blitzkrieg*’).

To see how this is done, consider that, for lithiated PCP, some of the surface chemical groups are electrochemically unstable (∼0.1 V versus E^0^_(Li^+^__/Li^0^__)_) and would be removed or reconfigured if the substrate is kept reducing enough and if a free electron can tunnel to the surface. This causes LiC_6_ itself to be lithiophilic (73°), which allows fast spreading of Li_liq_, driving out the oxidative gases quickly and plugging the pores, maintaining even more reducing ambience. Vice versa, successful oxidative defense would slow down the contact line and recruit more oxidative O_2_, CO_2_ to the contact lines and to the liquid-Li surfaces, which further slows down the spreading until the lines no longer move at all, while the open gas pores maintain percolation to the outside. In other words, both oxidative defense and reducing ‘*Blitzkrieg*’ attack dynamics are *self-reinforcing*. A successful ‘*Blitzkrieg*’ attack will reduce the nearby fibers to a more reducing potential (*U*∼0 V) to exceed the thermodynamic electrochemical-stability window of the surface chemical groups by solid-state diffusion *inside* the graphite, and covert these groups with free electrons before the mobile O_2_, CO_2_ gases can come in to form a passivating tunneling barrier, which will facilitate future *Blitzkrieg*. We also note that liquid-metal infiltration greatly improves the electronic percolation of the PCP as well.

In the graphite-powder experiment, mechanical stirring plays a crucial role such that, if the Li_2_O/Li_2_CO_3_ scales are broken and pure Li_liq_ can get into true contact with a graphite powder, Li intercalation occurs, leading to the phase change from C_6_ to LiC_6_ and complete envelopment of a LiC_6_ particle by Li_liq_ (can happen if ACA <90°), resulting in the random-walk dispersion and homogenous distribution of LiC_6_ particles in the Li matrix. On the other hand, without mechanical stirring, graphite powder floats on the liquid Li and intercalation is pretty slow.

To summarize, in the case of carbonaceous materials, we find that, with a more reducing electrode potential (lower *U*^α^), a more compact and cleaner initial surface (HOPG), mechanical agitation of Li_liq_ that breaks the surface oxide and lower *P*O_2_, *P*CO_2_ fugacities facilitate lithiophilicity and can even induce superlithiophilic behavior. On the other hand, with a dirtier and more tortuous initial surface (PCP), a more oxidative initial potential of the carbonaceous substrate and a higher *P*O_2_, *P*CO_2_ fugacities facilitate apparent lithiophobicity.

### Morphology evolution of Li–PCP composite

**Figure 3.**
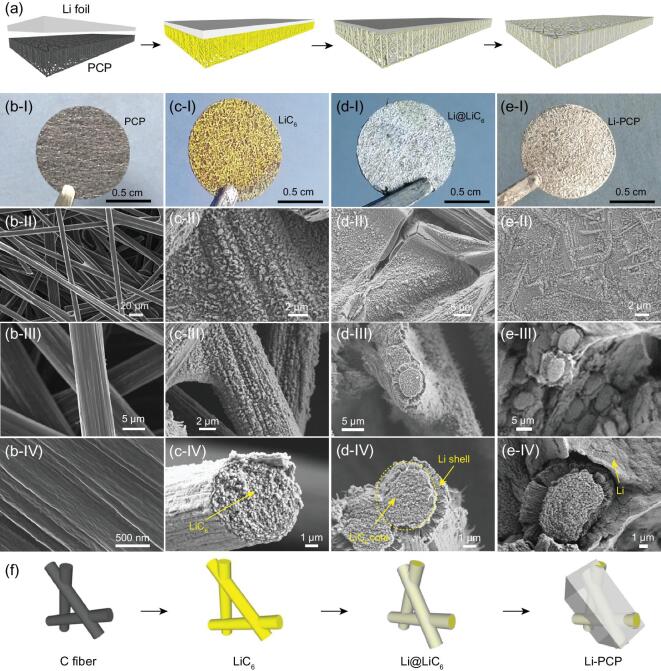
Structural evolution of the Li–PCP-composite fabrication process. (a) The schematic of the interaction between Li and PCP by adhering a piece of Li onto PCP upon heating. Photos and SEM images of (b) pristine PCP, (c) lithiated PCP, (d) Li@LiC_6_ core-shelled structure and (e) the final Li–PCP composite product. (f) The structural evolution schematic of graphitic fibers upon reacting with Li.

The fundamental understandings above enable us to develop a new approach to prepare self-standing Li–PCP composite. As illustrated in Fig. 3a, a piece of Li metal was pressed onto the surface of PCP and placed onto a hot plate in a glovebox. We can observe the significant color change of PCP from black to yellow and then metallic gray, demonstrating the successful formation of LiC_6_ and Li–PCP composite. Note that, according to our understanding, the mechanical pressing and the fact that the whole piece is collectively reduced assist the progress of the chemically reducing attack. The microstructural evolution was characterized by SEM. Figure 3b confirms that PCP was made of 1D fibers with an average diameter of 5 μm. The fiber surface is smooth and the whole structure is highly porous, with a porosity of 80%. Such a high porosity is favorable for storing large amounts of Li_BCC_ in the fabricated Li–PCP composite, if the oxidative ‘*Guerrilla warfare*’ defense can be overwhelmed and Li_liq_ can expel the vapor phase completely and shut off the percolating pores.

Li started to react with the PCP when the Li–PCP bilayer structure was placed on a hotplate and the color of the PCP turned to yellow (Fig. 3c). This corresponds to the formation of LiC_6_. SEM images in Fig. 3c show that the fiber surface became much rougher with numerous particles, although the whole fiber and porous structure were maintained well after the lithiation. According to our understanding, a freshly formed LiC_6_ is superlithiophilic [29]. With more Li incorporated, Li started to grow along the LiC_6_ fiber, forming a core–shell structure with LiC_6_ fiber as the core and excessive Li as the shell (Li@LiC_6_, Fig. 3d). In the final step, Li on the top layer was fully infused into the carbon framework and LiC_6_ fibers were embedded into the Li so that the fibers became fillers in the Li matrix (Fig. 3e). From the whole observations, we proposed the mechanism for the Li–PCP composite process: Li + PCP → LiC_6_ → core-shelled Li@LiC_6_ → Li*–*PCP composite (Fig. 3f). The transition from lithiophobicity to superlithiophilicity of PCP upon lithiation is attributed to the change in the carbon surface, with many pinning impurities converted into more reduced (metallic) and less potently pinning chemical groups. Due to the surface-chemistry change, the following Li infiltration can occur quickly, even in a *Blitzkrieg* fashion.

### Electrochemical performance of Li–PCP composite

**Figure 4.**
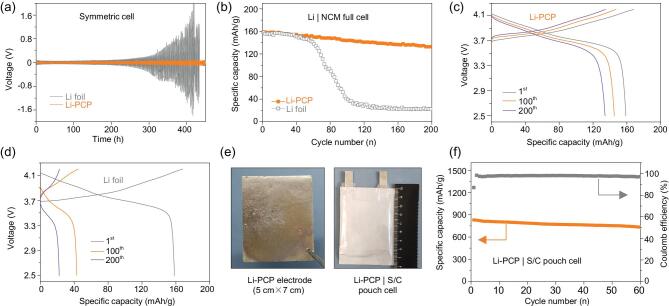
Electrochemical cycling performance of the Li–PCP electrode. (a) Voltage profiles of Li–PCP and Li-foil symmetric cells with a fixed capacity of 1.0 mAh/cm^2^ at 1.0 mA/cm^2^. (b) Cycling performance of Li–PCP/NCM and bare Li-foil/NCM cells at 0.5°C. Voltage profiles of (c) Li–PCP/NCM and (d) bare Li-foil/NCM at different cycles. (e) Digital images of a large piece of Li–PCP electrode and corresponding pouch cell with S/C cathode. (f) Cycling performance of the Li–PCP/S pouch cell.

Compared with the traditional methods of soaking or immersing PCP in a molten Li reservoir, the new approach can accurately control the weight ratio of Li in the Li–PCP composite, which is favorable for industrial-scale fabrication. To investigate the electrochemical performance of Li–PCP, here we paired it with different counter-electrodes. The first setup was using Li–PCP in Li/Li symmetric cells where identical Li–PCP electrodes were used to assemble coin cells within a common carbonate electrolyte (1.0 M LiPF_6_ in EC/DMC/EMC = 1:1:1 vol) while bare Li-foil cells were assembled as control. Figure 4a shows the representative voltage profiles of symmetric cells at 1.0 mA/cm^2^ with a fixed cycling capacity of 1.0 mAh/cm^2^. We see that the hysteresis voltage of the control cell gradually increased from 60 to 200 mV after 130 cycles, while the Li–PCP cell remained stable over 200 cycles. Supplementary Fig. 13 compares the zoomed-in voltage profiles at the first cycle and after 130 cycles, which demonstrates that the Li–PCP cell exhibits flat plating/stripping curves while the control cell cannot. When the cycling capacity was increased to 3.0 mAh/cm^2^, the control-cell voltage started to diverge after only 45 cycles while the Li–PCP cell cycled stably after 100 cycles with a slightly enlarged hysteresis voltage (Supplementary Fig. 14). The Li–PCP cell also showed a better rate performance than the control cell (Supplementary Fig. 15). We further compared the cycling performance of the Li–PCP and pure Li foil with an areal capacity of 3 mAh/cm^2^ at 3 mA/cm^2^, as shown in Supplementary Fig. 16. The voltage hysteresis of the symmetrical cell with the Li–PCP remained stable during the cycles, while that of the pure Li-foil cell increased rapidly. It is evident that Li–PCP composite has a better stability than bare Li foil, which is consistent with previous studies [30] that the conductive carbon framework could lower the local current density and the stable composite structure is favorable for addressing the volume-change issue upon repeated electrochemical plating/stripping.

The second setup was pairing Li–PCP with LiNi_0.5_Co_0.2_Mn_0.3_O_2_ (NCM) cathode to assemble full cells (Fig. 4b). The control bare Li-foil/NCM and Li–PCP/NCM cells show the same performance at the beginning, delivering specific capacities of about 150 mAh/g. However, the bare Li/NCM cell faded after about 40 cycles and showed capacity nosediving after about 60 cycles. In contrast, the Li–PCP/NCM cell can give a much better stability that the reversible capacity maintained at 130 mAh/g over 200 cycles. In Fig. 4c, it is clear that the

Li–PCP/NCM cell shows similar voltage profiles from the 1st cycle to the 200th cycle and delivers comparable capacities. In sharp contrast, a bare Li/NCM cell gives a large voltage polarization and a capacity as small as 25 mAh/g after 200 cycles (Fig. 4d). Due to the facile synthesis approach, a large piece of Li–PCP (5 × 7 cm^2^, Fig. 4e) has been successfully fabricated. A pouch-cell-type Li–PCP/Li_4_Ti_5_O_12_ (LTO) cell has been demonstrated, which shows long-term stability over 300 cycles while the control cell with bare Li metal fades after about 120 cycles (Supplementary Fig. 17). We further applied the large Li–PCP electrode in pouch-cell coupling with a sulfur/carbon (S/C) cathode. Here, a Li–PCP/S pouch cell with an areal capacity of ∼3.0 mAh/cm^2^ was assembled and showed a fairly stable cycle performance over 60 cycles at 0.5°C (Fig. 4f). In contrast, a bare Li_BCC_/S cell shows a rapid capacity decay (Supplementary Fig. 18). This proves the versatility of the Li–PCP electrode in different Li-metal batteries.

## CONCLUSION

In summary, graphite is intrinsically lithiophilic if we allow the substrate to be fully reduced (C_6_ → LiC_6_) with fewer disruptions by oxidizing agents (air). This was revealed by the small ACA of the Li droplet on HOPG, the rapid infiltration of liquid Li into PCP and the full envelopment of graphite powder by liquid Li, after the substrate is already fully lithiated and the potential drops to 0.05–0.1 V versus Li^+^/Li. On the other hand, if the substrate has a high redox potential (C_6_), surface-pinning sites reinforced by the constant new arrivals of a trace amount of oxidizing agents can completely impede the spreading of Li metal. The apparent lithiophilicity of any substrate is therefore hypothesized to be local-potential-dependent and ambient-vapor-partial-pressure-dependent. A fully dense Li–graphite composite anode can be prepared with a controlled Li/C ratio using a simple thermal treatment and exhibits great stability in full-cell batteries against NCM, LTO, S/C.

## METHODS

### Experimental evaluation of the wettability

All the experiments were conducted in a glovebox with both H_2_O and O_2_ concentrations below 0.1 ppm. Li foil was purchased from Tianjin Zhongneng Lithium Industry Co., Ltd. Before conducting the wetting experiment, impurities of lithium (mainly Li_2_O and Li_2_CO_3_) should be removed. The Li foil was first placed in a stainless-steel container on a hotplate at 225°C. After meltdown, a pair of stainless-steel tweezers were used to clamp the impurities away until the molten Li showed a smooth surface and looked shiny with a metal luster. Then the cleaned lithium droplets were transferred to the testing substrates, which were placed on a hotplate.

### Materials preparation

PCP was purchased from Toray Group (Toray paper 30, TGP-H-030). Before lithiated PCP preparation, the PCP was washed with diluted hydrochloric acid, deionized water and ethanol in sequence. Then the dried PCP was placed on the surface of the liquid lithium at 225°C and heated for several minutes. As soon as the PCP turned bright yellow, the lithiated PCP was separated from the liquid lithium.

For the preparation of the Li–PCP composite, we removed the impurities of the Li foil and then pressed it into the lithium foil in a glovebox. The clean lithium foil was soft and flexible. Thus, we were able to press the foil into the PCP with a Teflon roll. Then the lithium foil/PCP was placed on the hotplate (225°C). The weight ratio of the Li in the Li–PCP composite was ∼ 65% with a specific capacity of ∼2000 mAh/g_total_.

The graphite powder was purchased from Shanshan Technology (artificial graphite) without any further treatment. The Li–graphite-powder composite was prepared by mixing lithium foil and graphite powder in a stainless-steel container on a hotplate at 225°C. The graphite powder turned yellow first and gradually mixed evenly with the lithium with continuous stirring.

### *Ab initio* MD calculation

The *ab initio* MD calculations were performed using density functional theory, implemented in Vienna *ab initio* Simulation Package (VASP) [31,32]. The Perdew-Burke-Ernzerhof functional was adopted for the generalized gradient approximation (GGA) [33] to exchange-correlation potential. The energy cut-off was selected to be 460 eV.

The Crystallographic Information File (CIF) of the Li_BCC_ metal was downloaded from the website of Materialsproject.com. Based on the Li_BCC_ metal CIF file, a 3 × 3 × 3 super cell containing 54 Li atoms was created with Avogadro 1.2.0 [34], which was further converted into a POSCAR file via Pymatgen [35] (http://pymatgen.org). The POSCAR file, with an expanded system box of 22.2 × 25.6 × 32 Å, was used as the VASP input, which was large enough to form a droplet in the MD calculation. After 1000 MD steps performed at 1000 K, a desired Li-metal droplet was built.

The CIF of the graphite was downloaded from the Materialsproject.com website and then expanded to a super cell using two-layered graphene (432 C atoms) and a simulation box of 22.2 × 25.6 × 28 Å. After placing the as-prepared Li-metal droplet on top of the two-layered graphene with Pymatgen package, the system was run for 3000 steps at 500 K to achieve energy convergency. The atom trajectories of the last 1000 steps were dumped out as an XYZ file and then rendered with VMD 1.9.3 [36–41], from which the contact angle was approximately measured.

For the Li/LiC_6_ case, as the result shown in Supplementary Fig. 3, the *ab initio* MD calculation was conducted in the same way by replacing the two-layered graphene with a LiC_6_ super cell (300 C atoms and 50 Li atoms) created using the same method as in the Li/graphite case. The calculation process was the same as with the previous Li/graphite case.

### Materials characterization

A scanning electron microscope (sigma 300vp; Zeiss, Germany) operating at 3.0 kV was employed to characterize the morphology. A protect strategy was adopted to avoid the possible reactions between air-sensitive samples and the atmospheric environment by sealing them in an airtight container in a glovebox for the transfer to the SEM equipment. X-ray diffraction patterns were recorded on a Bruker D8 Advance diffractometer equipped with a Cu-Ka radiation source. XPS analysis was conducted on an American Thermo Fisher Scientific ESCALAB 50Xi to characterize the surface chemistry.

### Electrochemical measurement

Li/Li symmetric cells were assembled with Li–PCP or bare Li foil as both working and counter-electrodes. LTO electrodes were prepared by mixing LTO powder (80 wt%), carbon black (10 wt%) and PVDF (10 wt%) in N-methyl pyrrolidone (NMP) homogenously. The slurry was coated on copper foil and dried at 80°C overnight. For the NCM electrode, NCM523 powder (90%), carbon black (5%) and PVDF (5%) were mixed together and then coated on aluminum foil and dried at 80°C. As for the S/C electrode, sulfur (80%) and carbon black (20%) were first homogenously mixed and heated in a sealed can at 150°C. Then the obtained S/C mixture (80%) was further mixed with carbon black (10%) and LA-133 (10%). The slurry was cast onto the aluminum foil and dried at 50°C. All the electrodes were further roll pressed before being cut into circular disks.

All the coin cells were assembled with standard CR2025 coin-type in an Ar-filled glovebox with O_2_ and H_2_O content bellow 0.1 ppm. The active mass loadings of the NCM and LTO electrodes are 10 and 15 mg/cm^2^, respectively. A sulfur cathode with a sulfur loading of 2.5 mg/cm^2^ was used in the pouch cell. LiPF_6_ in ethylene carbonate (EC)/dimethyl carbonate (DMC)/ethylmethyl carbonate (EMC) (1.0 M; v/v/v = 1:1:1) was used as the electrolyte for the Li/Li symmetric cells, Li/LTO and Li/NCM cells. Lithium bis(trifluoromethanesulphonyl)imide (LiTFSI) in 1,3-dioxolane (DOL)/1,2-dimethoxyethane (DME) (1.0 M; v/v = 1:1) with 2% LiNO_3_ (DoDoChem) was employed as the electrolyte in the Li–S pouch cells. The negative/positive ratio (N/P ratio) in these full cells was ∼3. These coin cells and pouch cells were cycled in a Neware multichannel battery tester (CT-4000).

## Supplementary Material

nwz222_Supplemental_Files
