# Supplementary material for: Is graphite lithiophobic or lithiophilic?
Source: Natl Sci Rev. 2020 Jan 3;7(7):1208–17. doi: 10.1093/nsr/nwz222 (PMC8288999; doi:10.1093/nsr/nwz222)
Supplement: nwz222_Supplemental_Files [file nwz222_supplemental_files.zip › Revised_Supplementary_Is_graphite_lithiophobic_or_lithiophilic_R2.docx]

Supplementary Materials for

**Is graphite lithiophobic or lithiophilic?**

Jian Duan†, Yuheng Zheng†, Wei Luo*, Wangyan Wu, Tengrui Wang, Yong Xie, Sa Li, Ju Li* and Yunhui Huang*

*Correspondence to: weiluo@tongji.edu.cn (W.L.); liju@mit.edu (J.L.); and huangyh@tongji.edu.cn (Y.H.H.)

†Equal contribution

**This PDF file includes:**

Figs. S1 to S18

Captions for Movies S1 to S6


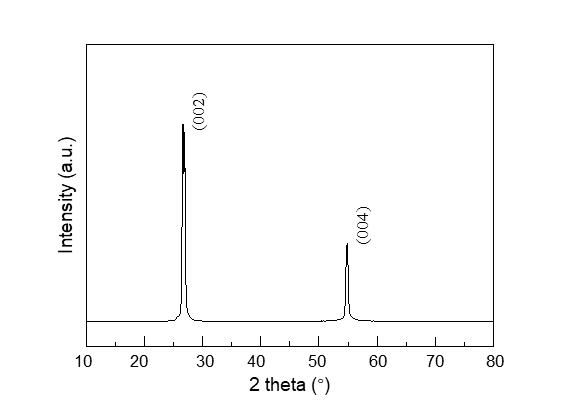


Fig. S1. X-ray diffraction pattern of the highly oriented pyrolytic graphite (HOPG).


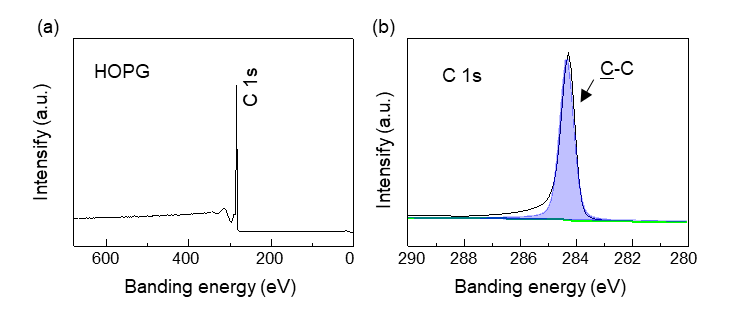


Fig. S2. Characterization of the surface chemistry of HOPG. (a) Survey XPS spectra; (b) High-resolution XPS spectrum of C1s for HOPG.


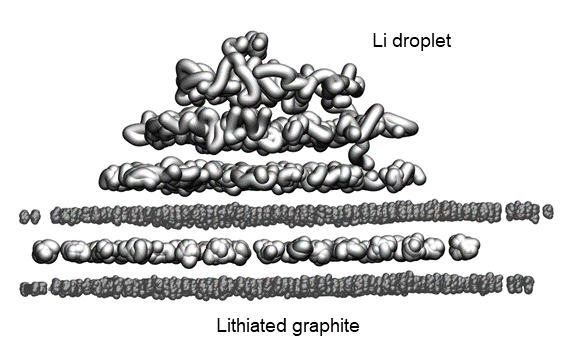


Fig. S3. Ab-MD calculation of a Li droplet/lithiated graphite system at 500K.


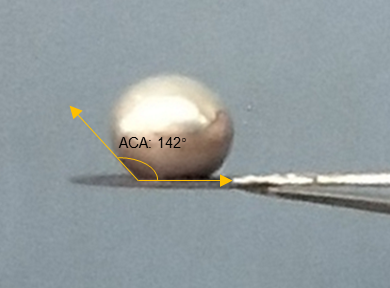


Fig. S4. Apparent contact angle (ACA) of lithium droplet on PCP. The ACA is as large as 142°, indicating PCP is lithiophobic.


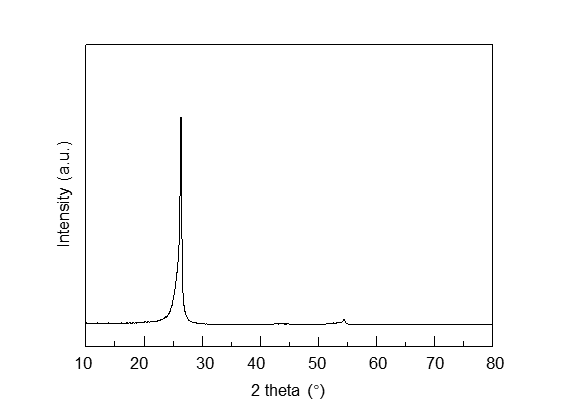


Fig. S5. X-ray diffraction pattern of PCP, showing it highly graphitic structure.


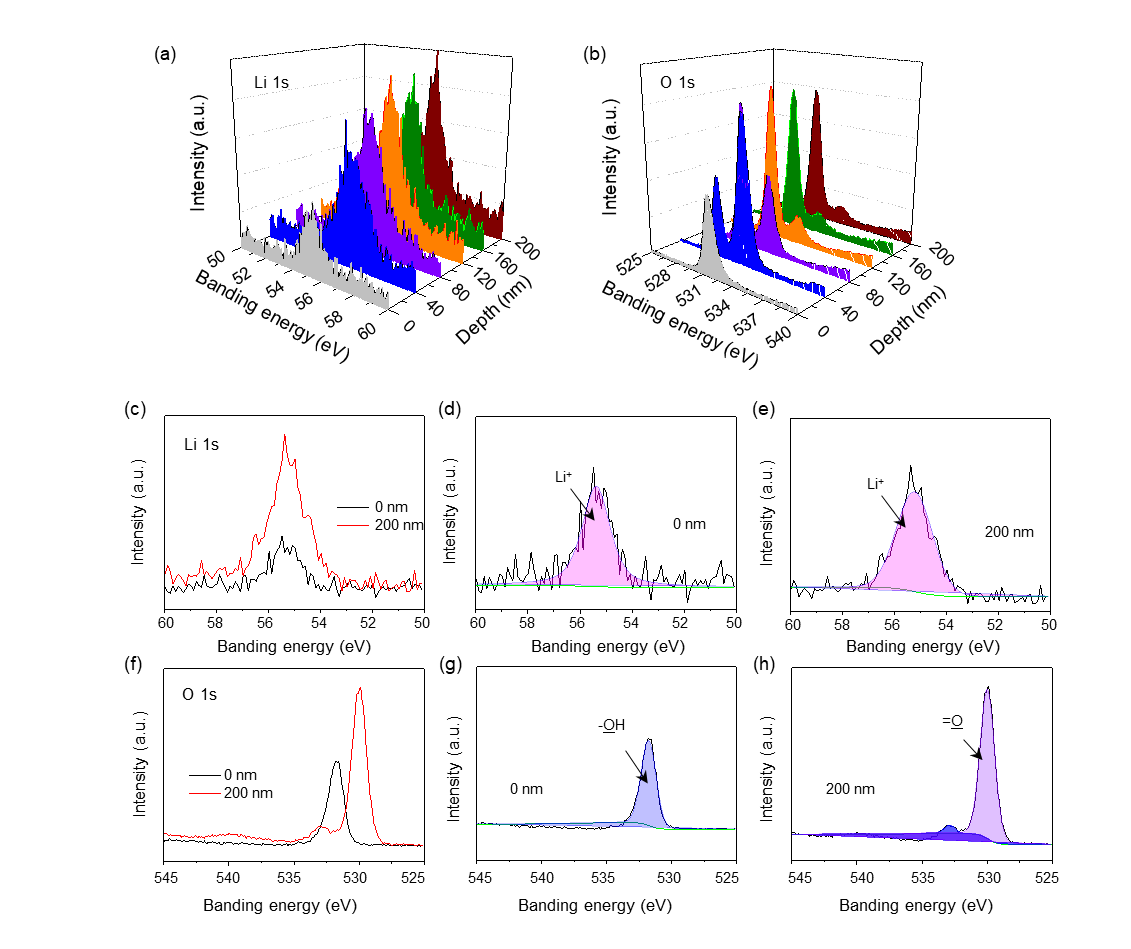


Fig. S6. XPS spectra of cleaned Li metal after storing in a glovebox for half an hour. Li 1s XPS spectra of cleaned Li metal at depth of (a) 0, 40, 80, 120, 160 and 200 nm, (c) 0 and 200 nm, (d) 0 nm and (e) 200 nm. O 1s XPS spectra of cleaned Li metal at depth of (b) 0, 40, 80, 120, 160 and 200 nm, (f) 0 and 200 nm, (g) 0 nm and (h) 200 nm.


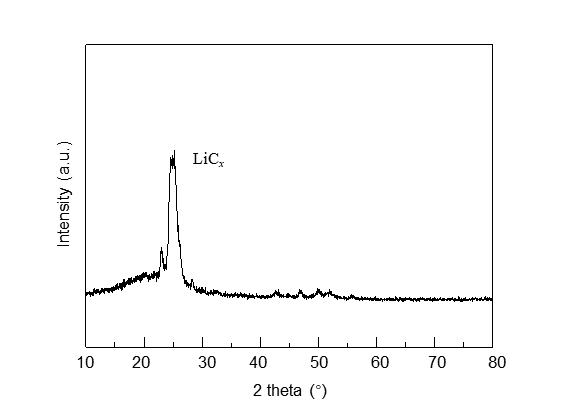


Fig. S7. X-ray diffraction pattern of lithiated PCP, showing the successful lithiation of the PCP.


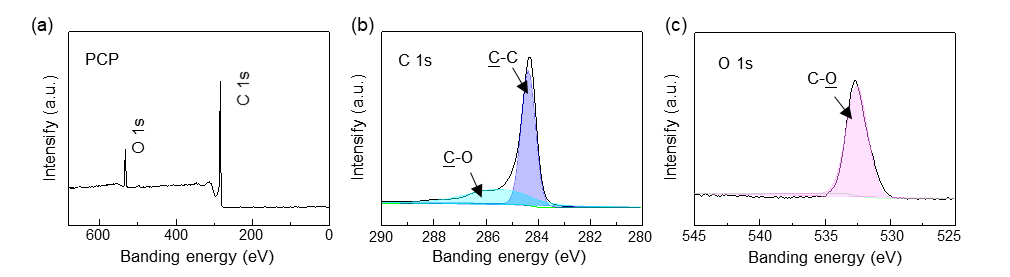


Fig. S8. Characterization of the surface chemistry of PCP. Survey XPS spectra (a) and high-resolution XPS spectrum of C1s (b) and O1s (c) for PCP.


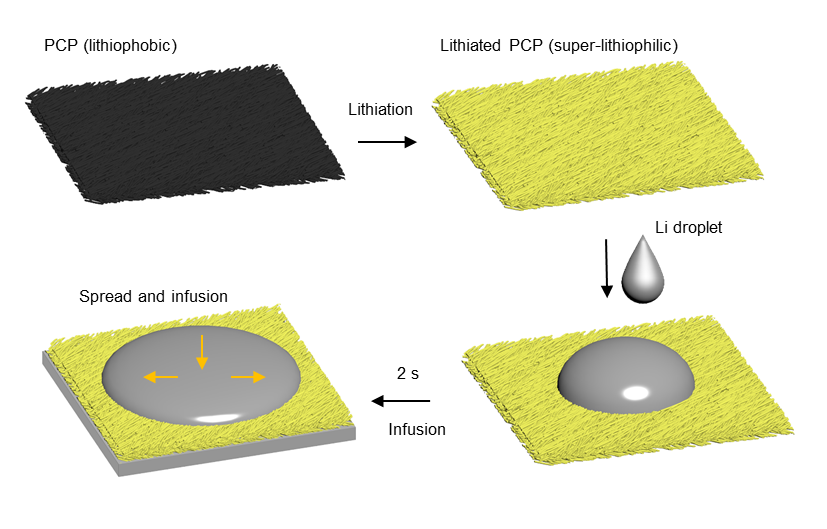


Fig. S9. Schematic of the transition process and infiltration of Li_liq_ into lithiated PCP.


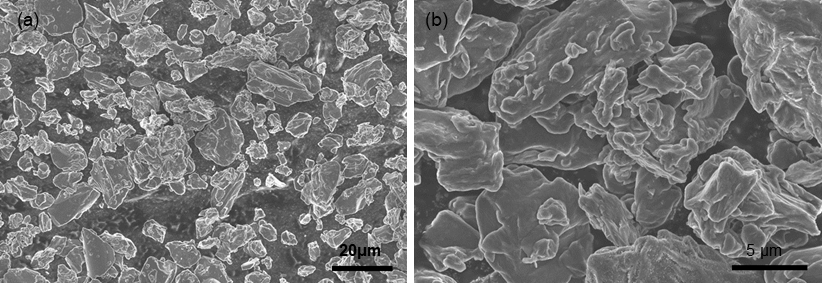


Fig. S10. SEM images of the graphite powder at different magnifications.


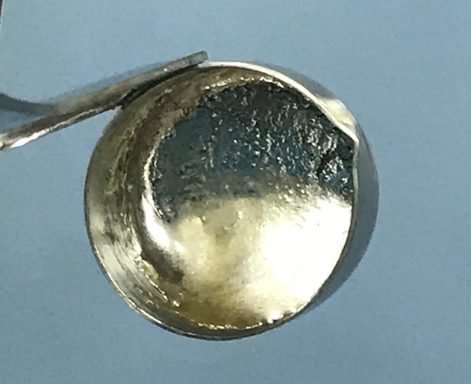


Fig. S11. Digital image of the Li_liq_-graphite powder composite.


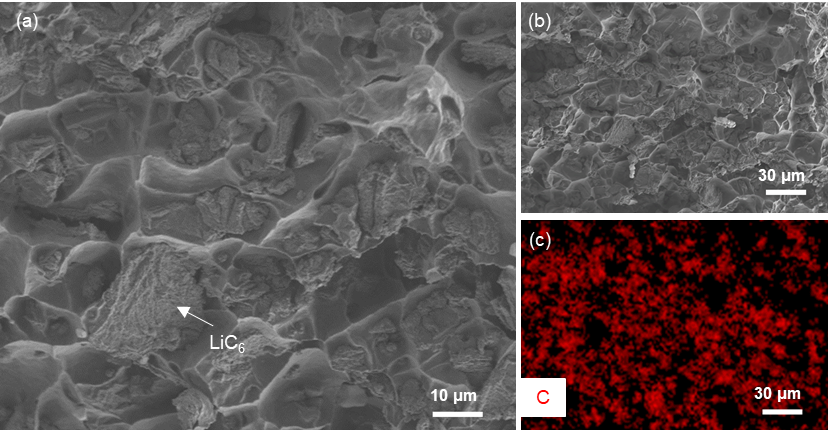


Fig. S12. (a, b) SEM and (c) corresponding EDX images of the Li-graphite powder composite.


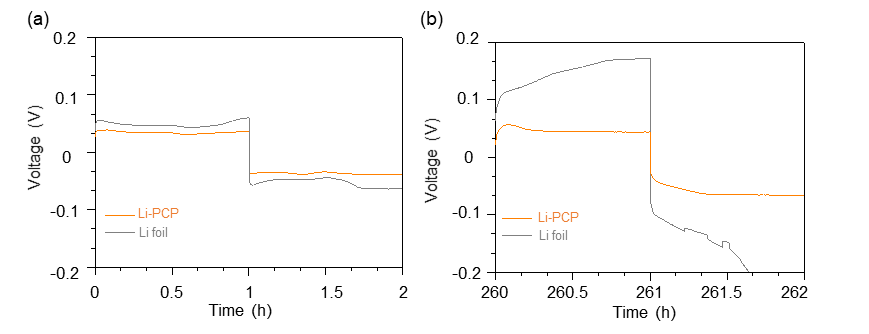


Fig. S13. Enlarged voltage profiles of (a) the first 2h and (b) the 260 – 262 h of the Li-PCP and Li foil symmetric cells at 1.0 mA/cm^2^.


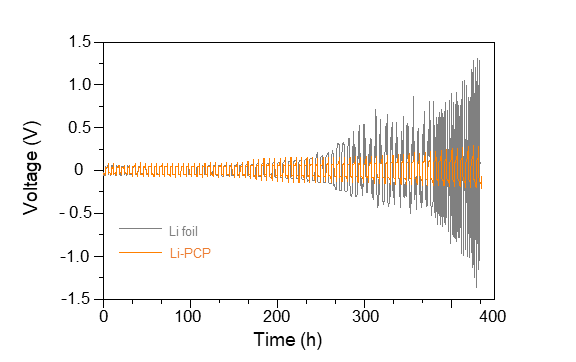


Fig. S14. Cycling performance of Li-PCP and Li foil symmetric cells at 1.0 mA/cm^2^ with a fix capacity of 3.0 mAh/cm^2^.

**
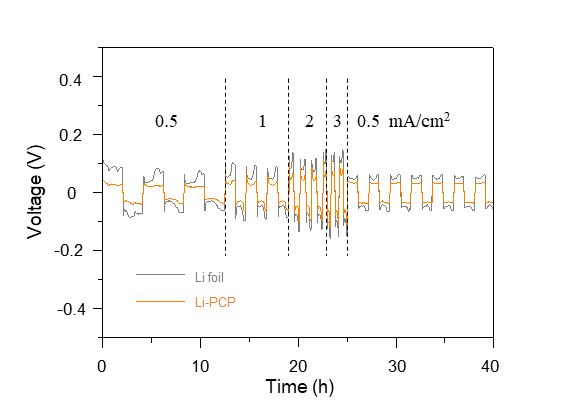
**

Fig. S15. Rate performance of Li-PCP and Li foil symmetric cells.

**
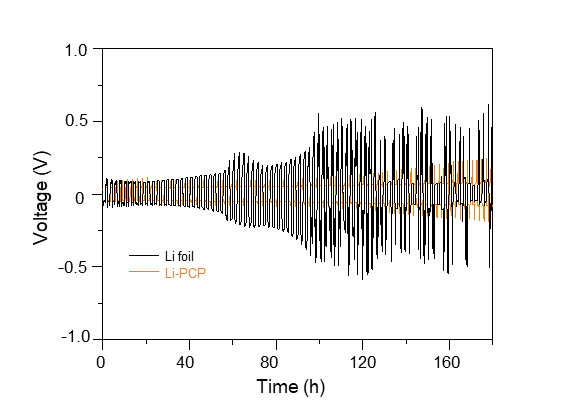
**

**Fig. S16.** Voltage profiles of symmetric cells with pristine Li foils or Li-PCP as electrodes tested with an areal capacity of 3 mAh/cm^2^ at 3 mA/cm^2^.


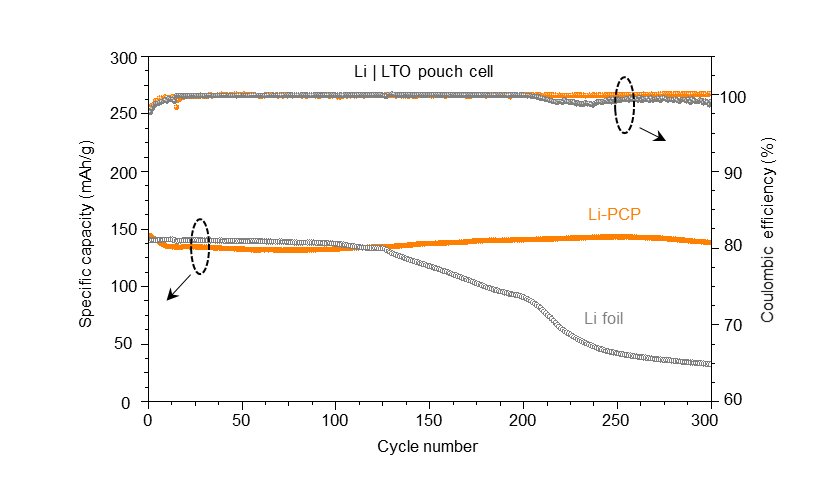


Fig. S17. Cycling performance of Li-PCP/LTO and Li foil/LTO pouch cell at 160 mA/g.

**
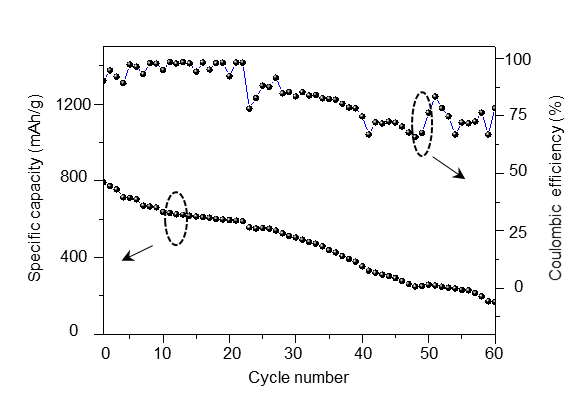
**

Fig. S18. Cycling performance of Li foil/S pouch cell at 0.5C.

Movie S1:

The apparent contact angle test between HOPG and liquid Li droplet shows that graphite is lithiophilic.

Movie S2:

The process of removing solidified Li droplet from HOPG, showing that lithium tightly adhered to HOPG.

Movie S3:

The apparent contact angle test between porous carbon paper (PCP) and liquid Li droplet shows that PCP shows lithiophobic. The liquid Li droplet tend to be “ball” on the surface of PCP with large apparent contact angle.

Movie S4:

The porous carbon paper (PCP) gradually turned to brilliant yellow during the synthesis process of the lithiated PCP.

Movie S5:

The apparent contact angle measurement of the lithiated PCP and lithium show that the lithiated PCP is superlithiophilic, indicating the pre-lithiated process is the key factor for the transformation of PCP from lithiophobicity to lithiophilicity.

Movie S6:

With the aid of mechanical stirring, the surface passivation of lithium is broken and the lithiated process of graphite powder is accelerated. Finally, a uniformly Li-graphite powder composite is obtained.
